# Dairy product consumption was associated with a lower likelihood of non-alcoholic fatty liver disease: A systematic review and meta-analysis

**DOI:** 10.3389/fnut.2023.1119118

**Published:** 2023-02-22

**Authors:** Wei Dai, Huiyuan Liu, Tingjing Zhang, Qing Chang, Yuhong Zhao, Chuanji Guo, Yang Xia

**Affiliations:** ^1^Department of Clinical Epidemiology, Shengjing Hospital of China Medical University, Shenyang, China; ^2^Key Laboratory of Precision Medical Research on Major Chronic Disease, Shenyang, China; ^3^School of Public Health, Wannan Medical College, Wuhu, China

**Keywords:** milk, non-alcoholic fatty liver disease, dairy, meta-analysis, systematic review

## Abstract

**Background and aims:**

Non-alcoholic fatty liver disease (NAFLD) is one of the most common causes of chronic liver disease. Several epidemiological studies attempted to assess the association between dairy product and the likelihood of NAFLD, but the contribution of dairy consumption to NAFLD remains controversial. We conducted a meta-analysis to investigate the association between dairy product consumption and NAFLD.

**Methods:**

We conducted a literature search using the PubMed, Web of Science and Scopus databases, we conducted a thorough search of the literature published before January 5, 2023. Combined odds ratios (ORs) and 95% confidence intervals (CIs) of NAFLD in relation to dairy product intake were estimated using random-effects models. Subgroup analysis and meta-regression were performed according to the study design, region, sex, body mass index (BMI), type of exposure, NAFLD diagnostic criteria, and exposure assessment tools.

**Results:**

We initially identified 4,634 relevant studies, of which 25 complied with the inclusion criteria, including seven cross-sectional studies, six case–control studies and one cohort study. A total of 51,476 participants (14,546 patients with NAFLD) were included in the meta-analysis. There was an inverse association between dairy product consumption and NAFLD (OR = 0.97, 95% CI = 0.94–0.99). Subgroup analysis demonstrated that lower likelihood of NAFLD was associated with dairy product consumption in subgroups of Asian populations, women, patients diagnosed using NAFLD-related scores, patients with a BMI of 18.5–24.9 kg/m^2^, dairy intake assessed using a food frequency questionnaire, milk consumption, and yogurt consumption. No noteworthy connection was observed in the other subgroups.

**Conclusion:**

Our meta-analysis findings revealed that dairy product consumption is inversely associated with NAFLD. Consumption of dairy products could help prevent the development of non-alcoholic fatty liver disease.

## Introduction

In the world today, non-alcoholic fatty liver disease (NAFLD) is rapidly becoming the most prevalent form of chronic liver disease (1), affecting approximately 25 and 10% of the global adult and pediatric populations, respectively (2, 3). NAFLD is defined by steatosis of at least 5% of hepatocytes, confirmed primarily by histology or high-resolution imaging, while excluding known hepatotoxic factors such as excessive alcohol consumption, viral infections, or illicit drug use (4). International guidelines suggest that lifestyle changes linked to nutrition should be a crucial component of NAFLD therapy, although there is no agreement on the pharmacological management of NAFLD (5). Therefore, it is necessary to identify healthy dietary interventions to reduce the burden of NAFLD.

Dietary guidelines worldwide recommend milk and dairy products (6), but it remains controversial whether dairy products are associated with NAFLD. Several recent studies have shown that dairy product consumption is not associated with NAFLD (7–9). In contrast, the results of a case–control study showed that dairy product consumption was associated with a lower likelihood of NAFLD (10). However, a cross-sectional study reported an increased likelihood of NAFLD associated with dairy consumption (11). Numerous unique fatty acids found in dairy products, including short-chain fatty acids and palmitoleic acid, which resemble hormones, may have beneficial metabolic effects (12, 13). In a previous study, dairy fat was found to enhance glucose tolerance, which may increase hepatic insulin sensitivity and systemically reduce liver fat deposition (14). However, the chances of developing NAFLD were increased by dairy products (especially cheese) in another study, which could be due to saturated fatty acids in dairy products (15). These inconsistent results highlight the diverse associations between different types of dairy intake and NAFLD.

To our knowledge, although a previous meta-analysis investigated the association between dairy intake and NAFLD, the inclusion of dairy product types was neither complete nor specific (e.g., ice cream was not included) (16). Therefore, we aimed to more accurately and comprehensively estimate the impact of dairy consumption, including that of various dairy products, on NAFLD. To achieve this, we assessed the association between the consumption of dairy products, and their different types, and the development of NAFLD. Furthermore, we conducted a subgroup analysis based on the included studies’ characteristics, such as study design, dietary assessment tools, and of the study populations and regions.

## Materials and methods

### Database searches

Using the PubMed, Web of Science and Scopus databases, we conducted a thorough search of the literature published before January 5, 2023 (CRD42022357457) to identify observational studies examining the association between dairy product consumption and NAFLD in adult patients (age ≥ 18 years). The following words were combined to find results (Supplementary Table 1): “dairy” or “total dairy” or “dairy product” or “dairy products” or “milk” or “whole milk” or “low-fat milk” or “full-fat milk” or “yogurt” or “yoghurt” or “cream” or “ice cream” or “cheese” or “butter” or “kefir” combined with “fatty liver” or “NAFLD” or “non-alcoholic fatty liver disease” or “hepatocellular cancer” or “hepatic cancer” or “steatohepatitis” or “steatosis” or “nonalcoholic steatohepatitis.” A flowchart of literature selection is presented, in accordance with the Preferred Reporting Items for Meta-Analysis (PRISMA) guidelines (17).

### Inclusion and exclusion criteria

De-duplication of identical documents from multiple databases through literature management software such as Endnote. Selection criteria for duplicate literature. (1) Selection of the one with the largest sample size. (2) Selection of the one with the longest follow-up period. (3) Select the one with the most comprehensive study outcomes. The following were the conditions for inclusion: (1) adult population; (2) a study that explored the association between dairy product consumption and the likelihood of developing NAFLD, such as cohort studies, case–control studies or cross-sectional studies; (3) a diagnosis of NAFLD made by ultrasound, magnetic resonance imaging (MRI), controlled attenuation parameter (CAP), fibro-scan, fatty liver index, or liver biopsy; (4) consumption of dairy products: total dairy, milk, yogurt, cheese, or other types of dairy products; (5) outcome was NAFLD; (6) language was restricted to English. Exclusion criteria were as follows: (1) teenaged or pregnant participants; (2) surface antigens for hepatitis B, antibodies against hepatitis C, or HIV antibodies present; (3) abnormally high alcohol or drug intake that could be hazardous to the liver (tamoxifen, steroids, and amiodarone); and (4) reviews, comments, editorials, letters, interviews, or reports.

### Data extraction and quality assessment

From each study, the following information was extracted: first author, country, publication year, study design, sample size, number of cases, body mass index (BMI), participants’ mean age, NAFLD diagnostic method, dietary assessment tools, types of exposure, risk estimate (HRs, RRs, or ORs and its 95% CI), and confounding factors adjusted in the final model. The Newcastle-Ottawa scale (18) and the Agency for Healthcare Research and Quality (19) were used to assess the quality of cohort studies, case–control studies, and cross-sectional studies. The Newcastle-Ottawa scale was used to rate the literature quality with a score out of 9 stars, with higher scores indicating higher quality studies; scores of ≥ 6 and < 6 indicated high- and low-quality studies, respectively. Scores of 0–3, 4–7 and 8–11 on the Agency for Healthcare Research and Quality indicated low-, moderate- and high-quality literature, respectively.

### Statistical analysis

The RRs and HRs used in this meta-analysis were assumed to be roughly equivalent to ORs. Heterogeneity was assessed using the *I*^2^ statistic (20), and *I*^2^ > 50% indicated a high degree of heterogeneity between the studies. Depending on the degree of heterogeneity, we either used fixed-effects or random-effects models to construct ORs and 95% CIs. (*I*^2^ < 50, fixed-effects model; *I*^2^ ≥ 50, random-effects model). After excluding each article from the overall analysis, a sensitivity analysis was conducted to determine how each article contributed to the overall composite result. To evaluate publication bias, Egger’s and Begg’s tests were used (21, 22). *p* < 0.05 was regarded as possibly biased due to publication.

In the subgroup analysis, the following factors were stratified: (1) study design (cross-sectional or case–control); (2) dietary assessment tools [food frequency questionnaire (FFQ) or dietary recall]; (3) regions (Europe or Asia); (4) sex (men or women); (5) BMI (18.5–24.9 kg/m^2^, ≥ 25 kg/m^2^, or ≥ 30 kg/m^2^); (6) types of exposure (milk, yogurt, cheese, or ice cream); and (7) NAFLD diagnostic criteria (CAP, ultrasonography, MRI, or NAFLD-related scores). Meta-regression analysis was conducted to determine possible associations between the above factors. Stata 17.0 software (Stata Corp, College Station, TX, United States) was used for the statistical analysis. In addition, the *p* value was two-tailed, with a value < 0.05 considered statistically significant.

## Results

### Literature search

**Figure 1:**
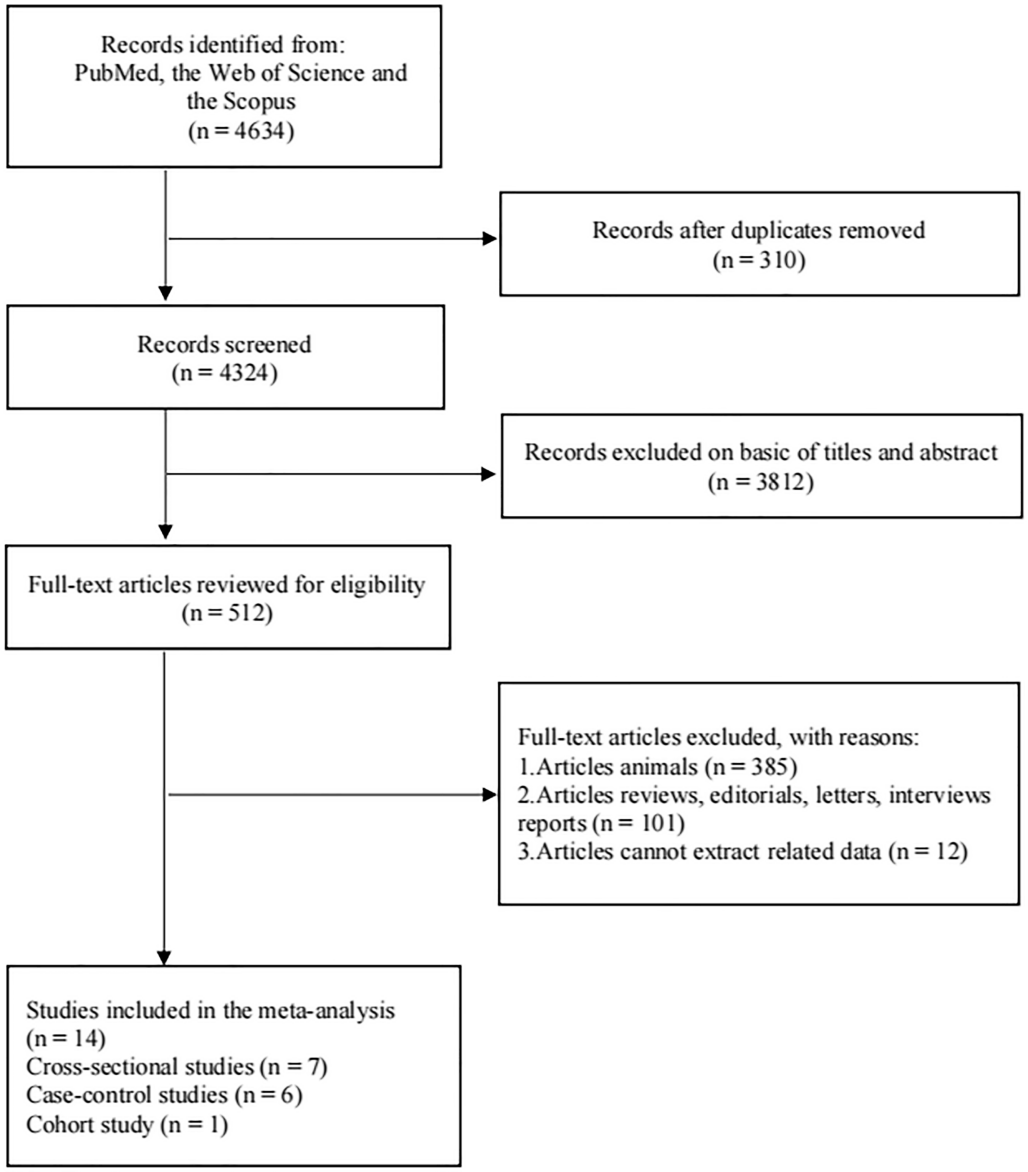
The flowchart of the study inclusion process.

A flowchart depicting the study selection process is shown in Figure 1. According to PubMed, Web of Science, and Scopus, 4,634 articles were identified. We excluded 310 articles that contained duplicate content and 3,812 articles for which the titles and abstracts did not match. The full text of the remaining 512 articles were read, and 498 were excluded because they were animal studies (*n* = 385), reviews or editorials (*n* = 101), or their data could not be extracted (*n* = 12). Ultimately, 14 studies (including seven cross-sectional studies, one cohort study and six case–control studies), including 25 estimates, met the inclusion criteria.

### Study characteristics

Table 1 summarizes the characteristics of the study. The inclusion criteria were met by a total of 14 studies with 51,476 participants (33,159 in seven cross-sectional studies, 5,171 in one cohort study and 13,146 in six case–control studies) and 14,546 cases of NAFLD (9,640 in seven cross-sectional studies, 1,799 in one cohort study and 3,107 in six case–control studies). There was a wide range of publication years, ranging from 2015 to 2022. Among them, four studies were from Europe and nine from Asia. The results were divided into different subgroups based on exposure type, including milk, yogurt, cheese, and ice cream.

**Table 1:** Characteristics of the included studies in this meta-analysis.

References	Country	Year	Study design	Sample size	Number of cases	BMI	Age	NAFLD diagnostic criteria	Dietary assessment tool	Exposures	OR/HR (95% CI)	Adjusted covariates
Chan et al. (23)	China	2015	Cross-sectional	797	220	25.5 ± 3.5	51.0 + 9.3	MRI	FFQ	Milk and products	1.53 (0.94–2.49)	Age, sex, BMI, current smoker status, current drinker status, central obesity, triglyceride >1.7 mmol/l, reduced HDL-cholesterol, hypertension, impaired fasting glucose, diabetes, and the PNPLA3 genotypes
Chiu et al. (24)	China	2018	Cross-sectional	3,400	1911	22.9 ± 3.0	55	Ultrasonography	FFQ	Dairy	1.02 (0.92–1.14)	Age, gender, education, history of smoking, history of alcohol drinking, total energy intake, vegetarian diet, and BMI
Charatcharoenwitth-aya et al. (25)	Switzer-land	2021	Cross-sectional	252	41	28.8 ± 4.5	40.9 ± 10.5	CAP	A food diary for seven consecutive days	Full-fat dairy	0.42 (0.18–0.99)	Age, sex, healthcare profession, and daily calorie intake
Hao et al. (26)	China	2021	Cross-sectional	4,049	2,602	26.9 ± 2.89	53.8 ± 7.46	Ultrasonography	A self-questionnaire	Milk and products	0.55 (0.43–0.70)	Age
Watzinger et al. (27)	German	2020	Cross-sectional	136	72	32.5 ± 3.6	50.1 ± 8.0	MRI	FFQ	High-fat dairy	0.32 (0.10–0.98)	Age, sex, WC, calorie intake, and the ratio of energy intake/total energy expenditure
										High-fat cheese	0.55 (0.17–1.76)	
Mirizzi et al. (11)	Italy	2019	Cross-sectional	136	136	33.41 ± 4.47	49.5 ± 10.1	CAP	FFQ	Milk and Yogurt	0.99 (0.99–1.00)	Age, sex, and energy intake
										Sweet Products Milk-Based	1.00 (0.97–1.03)	
										Aged Cheeses	0.98 (0.97–1.00)	
										Cheeses	0.99 (0.98–1.02)	
										Local Aged Cheeses	0.85 (0.74–0.98)	
										Sweet Milk-Nowinter Icecream	1.11 (1.01–1.21)	
										Industrial Aged Cheeses	1.17 (1.01–1.35)	
										Winter Ice cream	0.65 (0.47–0.89)	
Zhang et al. (28)	Tianjin, China	2019	Cross-sectional	24,389	4,658	27.8 ± 0.1	42	Ultrasonography	FFQ	Yogurt	0.86 (0.76–0.98)	Age, sex, BMI, smoking status, alcohol drinking status, education level, working status, household income, physical activity, family history of disease, total energy intake, carbohydrate intake, total fat intake, EPA + DHA intake, soft drinks intake, vegetables intake, fruits intake, sweet foods intake, milk intake; hypertension, diabetes, hyperlipidemia, and WBC count
Kalafati et al. (15)	Italy	2019	Case–control	351	134	31.11 ± 4.72	50.3 ± 10.5	Ultrasonography	FFQ	Cheese full fat	1.19 (1.00–1.42)	Age, gender, BMI, energy intake for the food groups, and pack-years
Ebrahimi et al. (7)	Iran	2022	Case–control	243	121	30.5 ± 5.0	42.9 ± 11.5	Ultrasonography	FFQ	Dairies	1.61 (0.71–3.63)	Age, sex, energy intake, physical activity, marital status, education, supplement use, drug use, smoking status, BMI, and DDS
Sun et al. (10)	China	2022	Case–control	11,888	2,529	23.7 ± 2.9	51.1 ± 14.6	Ultrasonography	FFQ	Dairy	0.85 (0.75–0.96)	/
Tutunchi et al. (9)	Iran	2021	Case–control	210	105	33.8 ± 7.7	45.6 ± 9.1	Ultrasonography	A three-day food diary	Low-fat dairy	0.46 (0.16–1.32)	/
										High-fat dairy	2.01 (0.81–3.11)	
Dehghanseresht et al. (29)	Iran	2020	Case–control	244	122	42.95 ± 11.46	30.53 ± 5.04	Ultrasonography	FFQ	Dairy	0.23 (0.09–0.58)	Age, sex, BMI, energy intake, smoking, educational status, and physical activity
Pasdar et al. (8)	Iran	2019	Case–control	210	96	30.49 ± 5.89	43.82 ± 8.79	Ultrasonography	FFQ	Dairy	1.75 (0.84–3.67)	Age, sex, and physical activity
Lee et al. (30)	Korean	2021	Cohort	5,171	1,799	23.5 ± 2.5	40–49	NAFLD liver fat score	FFQ	Milk	Men: 0.85 (0.73–0.99) Women ≥50 years: 0.79 (0.66–0.95) Women <50 years: 0.94 (0.76–1.16)	Age, BMI, physical activity, smoking status, current drinker, daily protein intake per weight, daily carbohydrate intake per weight, daily calcium intake, daily vitamin E intake, MBP, plasma glucose level, serum total cholesterol level, and serum ALT level
										Yogurt	Men: 0.91 (0.78–1.06) Women ≥50 years: 0.86 (0.73–1.01) Women <50 years: 0.97 (0.79–1.18)	

NAFLD, non-alcoholic fatty liver disease; CI, confidence interval; CAP, controlled attenuation parameter; FFQ, food frequency questionnaire; BMI, body mass index; DDS, dietary diversity score; MRI, magnetic resonance imaging; WBC, white blood cell; MBP, mean blood pressure; EPA, eicosapentaenoic acid; HDL high-density lipoprotein; DHA, docosahexaenoic acid; ALT, alanine aminotransferase; WC, waist circumference.

The calibration of the included studies is reported in Supplementary Tables 2 and 3. All studies were of appropriate quality, based on two scales. The quality scores of the cohort studies and case–control studies ranged from seven to eight, while those of the cross-sectional studies ranged from seven to nine.

### Association between dairy products consumption and NAFLD

**Figure 2:**
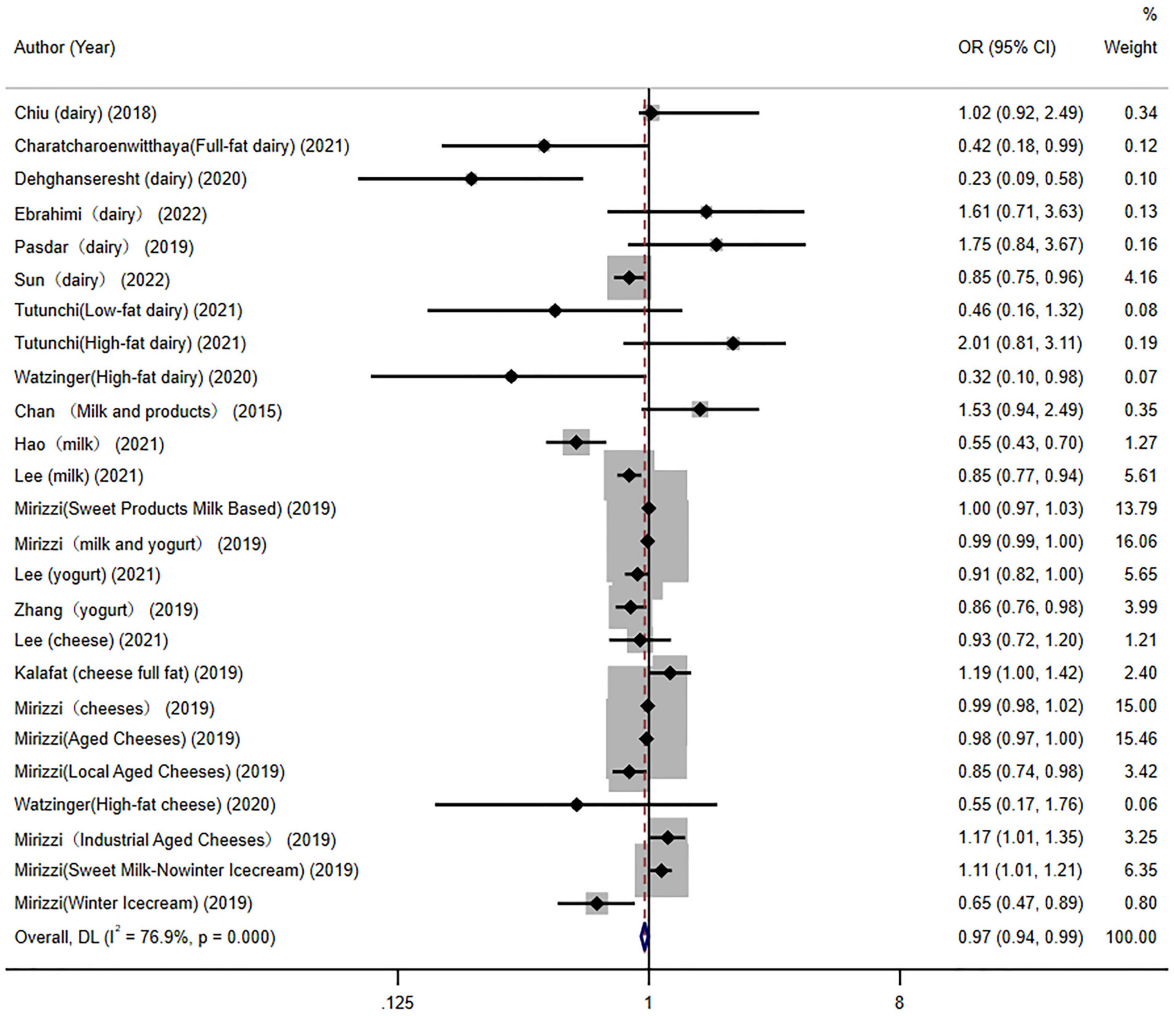
Overall pooled analysis of association between dairy products and non-alcoholic fatty liver disease.

In total, 14 articles (including 25 effect groups) investigated the associations between the consumption of dairy products and NAFLD; the results of the pooled analysis are shown in Figure 2. The forest plot showed that dairy product consumption was associated with a lower likelihood of NAFLD (OR = 0.97, 95% CI = 0.94–0.99).

### Subgroup analysis

Several subgroup analysis were also performed (Table 2). In accordance with, we divided the patients into two subgroups according to the design of each study (cross-sectional and case–control). The cross-sectional studies (OR = 0.98, 95% CI = 0.96–1.01) and case–control studies (OR = 1.02, 95% CI = 0.73–1.42) showed no significant association between dairy product consumption and NAFLD. Subgroup analysis stratified by sex demonstrated that dairy product consumption was associated with a lower likelihood of NAFLD (OR = 0.98, 95% CI = 0.95–0.99) in women, but not in men (OR = 0.83, 95% CI = 0.40–1.72). Subgroup analysis stratified by BMI demonstrated that dairy product consumption was associated with a lower likelihood of NAFLD in patients with a BMI of 18.5–24.9 kg/m^2^ (OR = 0.88, 95% CI = 0.83–0.93), but not in patients with a BMI ≥ 25 kg/m^2^ (OR = 0.78, 95% CI = 0.52–1.16) and a BMI ≥ 30 kg/m^2^ (OR = 1.00, 95% CI = 0.97–1.02). Based on the type of exposure, we divided all studies into four subgroups (milk, yogurt, cheese, or ice cream). The yogurt consumption (OR = 0.89, 95% CI = 0.82–0.96) and milk consumption (OR = 0.94, 95% CI = 0.87–1.00) was associated with a lower likelihood of NAFLD. However, cheese (OR = 0.99, 95% CI = 0.96–1.03), and ice cream (OR = 0.87, 95% CI = 0.52–1.47) were not associated with NAFLD. Subgroup analysis stratified by exposure assessment showed that consumption of dairy products assessed using FFQ was inversely associated with NAFLD (OR = 0.97, 95% CI = 0.94–0.99), while no significant association was found when exposure was assessed using dietary recall (OR = 0.76, 95% CI = 0.25–2.29). Subgroup analysis stratified by study region showed that dairy product intake was associated with a lower likelihood of NAFLD in Asian populations (OR = 0.90, 95% CI = 0.77–0.97), but not in European populations (OR = 0.99, 95% CI = 0.97–1.01). Finally, subgroup analysis stratified by assessments of NAFLD showed that dairy product intake was inversely associated with NAFLD when NAFLD-related scores (OR = 0.88, 95% CI = 0.83–0.95) were used; however, no significant association was found when NAFLD was assessed using ultrasonography (OR = 0.91, 95% CI = 0.73–1.13), MRI (OR = 0.72, 95% CI = 0.26–2.02), and CAP (OR = 0.99, 95% CI = 0.97–1.01).

**Table 2:** A subgroup analysis of the association between dairy product consumption and non-alcoholic fatty liver disease (NAFLD).

Subgroup	Number of effects	OR (95% CI)	*I*^2^ statistics (%)	Meta-regression (*P*)
*Study design*				0.26
Cross-sectional	15	0.98 (0.96, 1.01)	77.60%	
Case–control	7	1.02 (0.73, 1.42)	79.50%	
*Type of exposure*				0.47
Milk	5	0.94 (0.87, 1.00)	88.30%	
Yogurt	2	0.89 (0.82, 0.96)	0.00%	
Cheese	7	0.99 (0.96, 1.03)	62.70%	
Ice cream	2	0.87 (0.52, 1.47)	90.00%	
*Region*				0.04
Europe	11	0.99 (0.97, 1.01)	70.70%	
Asia	12	0.90 (0.77, 0.97)	66.80%	
*Sex*				0.43
Men	20	0.83 (0.40, 1.72)	78.50%	
Women	5	0.98 (0.95, 0.99)	80.30%	
*BMI*				0.01
18.5–24.9	5	0.88 (0.83, 0.93)	0.00%	
≥ 25	4	0.78 (0.52, 1.16)	84.30%	
≥ 30	16	1.00 (0.97, 1.02)	71.70%	
*Exposure assessment*				0.91
FFQ	22	0.97 (0.94, 0.99)	77.60%	
Dietary recall	3	0.76 (0.25, 2.29)	80.20%	
*NAFLD diagnostic criteria*				0.03
CAP	9	0.99 (0.97, 1.01)	71.90%	
Ultrasonography	10	0.91 (0.73, 1.13)	78.60%	
MRI	3	0.72 (0.26, 2.02)	73.70%	
Score^a^	3	0.88 (0.83, 0.95)	0.00%	

a Score used by NAFLD liver fat score (LFS).

OR, odds ratio; CI, confidence interval; CAP, controlled attenuation parameter; BMI, body mass index; FFQ, food frequency questionnaire; MRI, magnetic resonance imaging.

### Sensitivity analysis and meta-regression

When successive articles were excluded from the sensitivity analysis, the results remained unchanged (Supplementary Figure 1). Meta-regression analysis (Table 2) showed that study design (*p* = 0.26), type of exposure (*p* = 0.47), sex (*p* = 0.43), and exposure assessment (*p* = 0.91) were not associated with heterogeneity, while region, NAFLD diagnostic criteria and BMI had a significant effect on heterogeneity (*p* < 0.05).

### Publication bias

Publication bias was evaluated using Egger’s test (*p* > 0.05), Begg’s test (*p* > 0.05), and funnel plots (Supplementary Figure 2), and these analysis revealed no publication bias.

## Discussion

The results showed that a lower likelihood of NAFLD was associated with dairy product consumption. However, when the types of dairy products were categorized, yogurt consumption and milk consumption were found to be significant among the factors associated with a lower likelihood of NAFLD. Subgroup analysis suggested that dairy product consumption was linked to a lower likelihood of NAFLD in subgroups of Asian populations, women, patients diagnosed using NAFLD-related scores, patients with a BMI of 18.5–24.9 kg/m^2^, and dairy product intake assessment using FFQ. In contrast, no significant associations were observed in the other subgroups. To explore possible sources of (expected) heterogeneity in the studies, we performed subgroup and meta-regression analysis, used sensitivity analysis to check the robustness of the results, and measured publication bias. Significant heterogeneity was present in most analysis, and this significant heterogeneity may reflect differences in the characteristics of the study population (region and BMI) and in the diagnostic approach to NAFLD. In a previous study, European populations were identified as a possible source of heterogeneity (31), which may be related to regional differences in the inclusion of participants. In addition, there were other factors such as study design and sample size that may also affect heterogeneity.

A previous study evaluated the association between dairy product intake and NAFLD (16), finding no significant correlation in cross-sectional studies. However, based on the results of case–control studies, dairy consumption was found to be positively associated with NAFLD. Despite this, as a result of the small sample size, the results of this previous study were limited. Moreover, the associations between dairy products and NAFLD in the different subgroups were not explored. Therefore, it was not possible to conclude from this previous study that dairy products caused NAFLD in a comprehensive manner. In this study, compared to previous meta-analysis, firstly, we conducted an updated search with more stringent inclusion criteria; whereas the previous meta-analysis was conducted in 2019, we conducted a thorough search for literature published before 5 January 2023. Secondly, ten additional articles were included in comparison to the previous article. Third, we included a more comprehensive range of dairy product types (e.g., ice cream) Fourth, we performed subgroup analysis and meta-regression based on study design, region, sex, BMI, type of exposure, NAFLD diagnostic criteria and exposure assessment tools. Fifth, we also performed a sensitivity analysis simultaneously. An analysis of 96 patients revealed that low-fat dairy intake was negatively correlated with NAFLD (32), a finding supported by a prospective cohort study of 101,510 Chinese participants demonstrating lower likelihood of NAFLD risk among consumers of dairy products (10). Several possible mechanisms could explain why dairy product intake is negatively associated with NAFLD. First, dairy products offer crucial dietary micronutrients such as calcium, iron, and vitamins. (33). A higher intake of dairy products may help prevent skeletal sarcopenia, an established risk factor for NAFLD (34). Second, diabetes-related insulin resistance is a major cause of NAFLD. An epidemiological study showed that dairy intake is negatively correlated with insulin resistance (35). Third, dairy consumption, particularly low-fat dairy products, had positive benefits on insulin resistance, waist circumference, and body weight, according to a recent meta-analysis (36), which was beneficial for NAFLD.

Our results found a negative association between yoghurt consumption and NAFLD. In line with our findings, a cross-sectional study of 24,389 adults found that participants who consumed yoghurt more than four times a week were less likely to develop NAFLD. Other similar studies have shown that yogurt consumption may have a preventive role in the development of other chronic diseases (28). The following aspects may partially explain the observed results. First, probiotics are abundant in yogurt. Probiotics may prevent the onset of NAFLD by inhibiting the lipopolysaccharide and hepatic toll-like receptor 4 signaling pathway, according to animal studies (37). Second, previous research has shown that probiotics from yogurt have anti-inflammatory, antioxidant, and immune-modulating properties, which may explain why people who consume more yogurt have a reduced prevalence of NAFLD (38, 39). Finally, yogurt is one of the most nutrient-dense foods and is high in proteins, vitamins, and minerals (such as calcium, magnesium, and potassium). There is proof that consuming more calcium, which is present in yogurt, results in more fat being burned throughout the body (40).

Furthermore, our study found that dairy product intake was inversely associated with NAFLD in Asian but not European populations, which may be due to differences in the epidemiological features of NAFLD and the dairy consumption habits = in different regions. For example, cheese, which has a high saturated fatty acid content, is much more popular in European populations than in Asian populations. We also found a clear positive correlation between dairy intake and NAFLD in women, but not in men. Previous studies (41) have shown that dairy product consumption was more protective against NAFLD in women than in men. This may be because estradiol exerts a protective effect against liver injury by inhibiting lipid accumulation and liver fibrosis (42). Lifestyle changed, including dietary habits and physical activity, should be the first line of treatment for NAFLD. Dietary modification therefore played a key role, as diets rich in carbohydrates, especially those high in fructose, were a major cause of obesity, insulin resistance and the development of NAFLD (43). Following the Mediterranean diet can reduce liver fat, even without weight loss, and it is the most recommended dietary pattern for NAFLD. The Mediterranean diet is characterized by a reduced intake of carbohydrates, especially sugar and refined carbohydrates, and an increased intake of monounsaturated and omega-3 fatty acids (44). The Mediterranean diet has also been found to improve metabolism and lower the risk of diabetes (45) and cardiovascular disease (46), two outcomes that are closely associated in people with NAFLD.

This study had several advantages. First, we conducted a comprehensive systematic search and applied comprehensive subgroup analysis to assess the associations between dairy consumption, including that of various dairy products, and NAFLD. Second, sensitivity and meta-regression analysis were performed to check the robustness of the results and explore potential heterogeneity.

However, the study also had several limitations. First, few articles were included in this study, and data on American populations were especially lacking. Second, all the included studies were observational, which has inherent limitations; for example, causality was uncertain. Third, the majority of patients with NAFLD in the included studies were diagnosed using ultrasonography, which is not the gold standard diagnostic method. However, liver biopsy is only performed when clinically indicated and is neither practical nor ethical for large epidemiological studies. Fourth, doogh is an important dairy product in parts of Asia, but we found no evidence for it in the available evidence, so further research is needed to explore this topic.

## Conclusion

In conclusion, a lower likelihood of NAFLD was associated with dairy product consumption, particularly milk consumption and yogurt consumption. Consumption of dairy products could help prevent the development of non-alcoholic fatty liver disease. However, given the few studies included, the results need further confirmation by more cohort studies and randomized controlled trials.

## Data availability statement

The original contributions presented in the study are included in the article/Supplementary material, further inquiries can be directed to the corresponding authors.

## Author contributions

WD and HL: conceptualization, formal analysis, visualization, and writing—original draft. TZ, QC, YZ, and CG: writing—review and editing. YX: conceptualization, resources, writing—review and editing, supervision, and funding acquisition. All authors contributed to the article and approved the submitted version.

## Funding

This study was supported by the National Natural Science Foundation of China (grant number: 81903302), the Young Elite Scientists Sponsorship Program by China Association for Science and Technology (grant number: YESS20200151), and the 345 Talent Project of ShengJing Hospital of China Medical University (grant number: M0294).

## Conflict of interest

The authors declare that the research was conducted in the absence of any commercial or financial relationships that could be construed as a potential conflict of interest.

## Publisher’s note

All claims expressed in this article are solely those of the authors and do not necessarily represent those of their affiliated organizations, or those of the publisher, the editors and the reviewers. Any product that may be evaluated in this article, or claim that may be made by its manufacturer, is not guaranteed or endorsed by the publisher.

## Abbreviations

NAFLD: non-alcoholic fatty liver disease
CIs: confidence intervals
ORs: odds ratios
RRs: relative risks
BMI: body mass index
CAP: controlled attenuation parameter
FFQ: food frequency questionnaire
MRI: magnetic resonance imaging
