# Supplementary material for: Dairy product consumption was associated with a lower likelihood of non-alcoholic fatty liver disease: A systematic review and meta-analysis
Source: Front Nutr. 2023 Feb 22;10:1119118. doi: 10.3389/fnut.2023.1119118 (PMC9992538; doi:10.3389/fnut.2023.1119118)
Supplement: Supplementary file 1 [file Data_Sheet_1.docx]

**Table S1. Assessment scale of cohort study or case-control study ^a^**

| Study | Selection | | | | Comparability | Outcome | | | Quality  score |
| --- | --- | --- | --- | --- | --- | --- | --- | --- | --- |
|  | Representativeness of the exposed cohort | Selection of the non-exposed cohort | Ascertainment of exposure | Demonstration that the outcome of interest was not present at start of the study | Comparability of cohorts on the basis of the design or the analysis | Ascertainment of outcome | Was follow-up long enough for outcomes to occur? | Adequacy of follow-up of cohorts |  |
| **Kalafati (2019)** | * | * | * | * | * | * | * | / | 7 |
| **Ebrahimi (2022)** | * | * | / | * | ** | * | * | / | 7 |
| **Sun (2022)** | * | * | / | * | ** | * | * | / | 7 |
| **Tutunchi (2021)** | * | * | * | * | ** | * | * | / | 8 |
| **Dehghanseresht (2020)** | * | * | * | * | ** | * | * | / | 8 |
| **Pasdar (2019)** | * | * | / | * | ** | * | * | / | 7 |
| **Lee (2021)** | * | * | * | * | ** | * | * | / | 8 |

^a^ The Newcastle–Ottawa Scale was used to assess the quality of studies

**Table S2. Cross-sectional study quality assessment scale ^a^**

| Studies | QA tool criteria | | | | | | | | | | | Quality score |
| --- | --- | --- | --- | --- | --- | --- | --- | --- | --- | --- | --- | --- |
| Reference | 1 | 2 | 3 | 4 | 5 | 6 | 7 | 8 | 9 | 10 | 11 |  |
| **Chan (2015)** | Yes | Yes | Yes | Yes | Yes | Unclear | Unclear | Yes | Yes | Yes | No | 8 |
| **Chiu (2018)** | Yes | Yes | Yes | Yes | Yes | Unclear | Yes | Yes | Yes | Yes | Unclear | 9 |
| **Charatcharoenwitthaya (2021)** | Yes | Yes | Yes | Yes | Yes | No | Unclear | Yes | Unclear | Yes | No | 7 |
| **Hao (2021)** | Yes | Yes | Yes | Yes | Yes | Unclear | Unclear | Yes | Yes | Yes | Unclear | 8 |
| **Watzinger (2020)** | Yes | Yes | Yes | Yes | Yes | Unclear | Yes | Yes | Unclear | Yes | Unclear | 8 |
| **Mirizzi (2019)** | Yes | Yes | Yes | Yes | Yes | Unclear | Yes | Yes | Unclear | Yes | Unclear | 8 |
| **Zhang (2019)** | Yes | Yes | Yes | Yes | Yes | Unclear | Yes | Yes | Yes | Yes | Unclear | 9 |

^a^ The Agency for Healthcare Research and Quality was used to assess the quality of studies

*1. Define the source of information (survey, record review)

2. List inclusion and exclusion criteria for exposed and unexposed subjects (cases and controls) or refer to previous publications

3. Indicate time period used for identifying patients

4. Indicate whether or not subjects were consecutive if not population-based

5. Indicate if evaluators of subjective components of study were masked to other aspects of the status of the participants

6. Describe any assessments undertaken for quality assurance purposes (e.g., test/retest of primary outcome measurements)

7. Explain any patient exclusions from analysis

8. Describe how confounding was assessed and/or controlled

9. If applicable, explain how missing data were handled in the analysis

10. Summarize patient response rates and completeness of data collection

11. Clarify what follow-up, if any, was expected and the percentage of patients for which incomplete data or follow-up was obtained

**Table S3. Meta-analysis search strategy.**

| **Database** | **Search strategy** |
| --- | --- |
| **Pubmed** | ("Dairy Products"[Mesh]) OR (Dairy Product)) OR (Product, Dairy)) OR (Products, Dairy)) OR (milk product)) OR (dairy produce)) OR (milk powder)) OR (powdered milk)) OR (dried milk)) OR (whole milk)) OR (low-fat milk)) OR (skimmed milk)) OR (at-free milk)) OR (yogurt)) OR (yoghurt)) OR (cream cake)) OR (ice cream)) OR (cheese)) OR (condensed milk)) OR (concentrated milk)) OR (milk shake)) OR (butter)) OR (milk)) OR (sherbet)) OR (cream)) OR (total dairy)) OR (frozen desserts)) OR (dairy)) OR (dairy product consumption)) OR (dairy product intake)) OR (dairy intake)) OR (fermented milk)) OR (kefir)) OR (low-fat dairy)) OR (low-fat total dairy)) OR (full-fat total dairy)) OR (full-fat milk)) AND (((("Liver Neoplasms"[Mesh]) OR (Neoplasms, Hepatic) OR (Neoplasms, Liver)) OR (Liver Neoplasm)) OR (Neoplasm, Liver)) OR (Hepatic Neoplasms)) OR (Hepatic Neoplasm)) OR (Neoplasm, Hepatic)) OR (Hepatocellular Cancer)) OR (Cancers, Hepatocellular)) OR (Hepatocellular Cancers)) OR (Hepatic Cancer)) OR (Cancer, Hepatic)) OR (Cancers, Hepatic)) OR (Hepatic Cancers)) OR (Cancer, Hepatocellular))) OR (("Non-alcoholic Fatty Liver Disease"[Mesh]) OR (Non alcoholic Fatty Liver Disease) OR (NAFLD)) OR (Nonalcoholic Fatty Liver Disease)) OR (Fatty Liver, Nonalcoholic)) OR (Fatty Livers, Nonalcoholic)) OR (Liver, Nonalcoholic Fatty)) OR (Livers, Nonalcoholic Fatty)) OR (Nonalcoholic Fatty Liver)) OR (Nonalcoholic Fatty Livers)) OR (Nonalcoholic Steatohepatitis)) OR (fatty liver) OR (Nonalcoholic Steatohepatitides)) OR (Steatohepatitides, Nonalcoholic)) OR (Steatohepatitis, Nonalcoholic) OR (Steatohepatitis, Nonalcoholic) |
| Web of Science | ＃1: TS=（Non alcoholic Fatty Liver Disease OR Nonalcoholic Fatty Liver Disease OR Fatty Liver, Nonalcoholic OR Fatty Livers, Nonalcoholic OR Liver, Nonalcoholic Fatty OR Livers, Nonalcoholic Fatty OR Nonalcoholic Fatty Liver OR Nonalcoholic Fatty Livers OR Nonalcoholic Steatohepatitis OR Nonalcoholic Steatohepatitides OR Steatohepatitides, Nonalcoholic OR Steatohepatitis, Nonalcoholic OR liver neoplasms OR Neoplasms, Hepatic OR Neoplasms, Liver OR Liver Neoplasm OR Neoplasm, Liver OR Hepatic Neoplasms OR Hepatic Neoplasm OR Neoplasm, Hepatic OR Hepatocellular Cancer OR fatty liver OR Cancers, Hepatocellular OR Hepatocellular Cancers OR Hepatic Cancer OR Cancer, Hepatic OR Cancers, Hepatic OR Hepatic Cancers）  ＃2: TS=(Dairy Products OR Dairy Product OR Product, Dairy OR Products, Dairy OR milk product OR dairy produce OR milk powder OR powdered milk OR dried milk OR whole milk OR low-fat milk OR skimmed milk OR fat-free milk OR yogurt OR yoghurt OR cream OR cream cake OR ice cream OR cheese OR condensed milk OR concentrated milk OR milk shake OR butter OR milk OR sherbet OR total dairy OR frozen desserts OR dairy OR dairy product consumption OR dairy product intake OR dairy intake OR fermented milk OR kefir OR low-fat dairy OR low-fat total dairy OR full-fat total dairy OR full-fat milk)  ＃3:＃1 and ＃2 |
| Scopus | ＃1: {dairy product} OR {milk} OR {yogurt*} OR {cream*} OR {cheese} OR {butter} OR {sherbet} OR {kefir} OR "total dairy" OR "ice cream" OR "low-fat dairy" OR "full-fat total dairy" OR "full-fat milk" OR "milk powder" OR "cream cake" OR "whole milk" OR "frozen desserts": TITLE-ABS-KEY＃2: {nafld} OR "nonalcoholic fatty liver disease" OR "nonalcoholic steatohepatitis" OR "liver neoplasms" OR "hepatic steatosis" OR {nash} OR "hepatic cancer*" OR "fatty liver" OR "liver neoplasm" OR "hepatic neoplasms" OR "hepatic cancer" OR "hepatocellular cancer": TITLE-ABS-KEY ＃3:＃1 and ＃2 |
