# Supplementary figures and images for: Dairy product consumption was associated with a lower likelihood of non-alcoholic fatty liver disease: A systematic review and meta-analysis
Source: Front Nutr. 2023 Feb 22;10:1119118. doi: 10.3389/fnut.2023.1119118 (PMC9992538; doi:10.3389/fnut.2023.1119118)

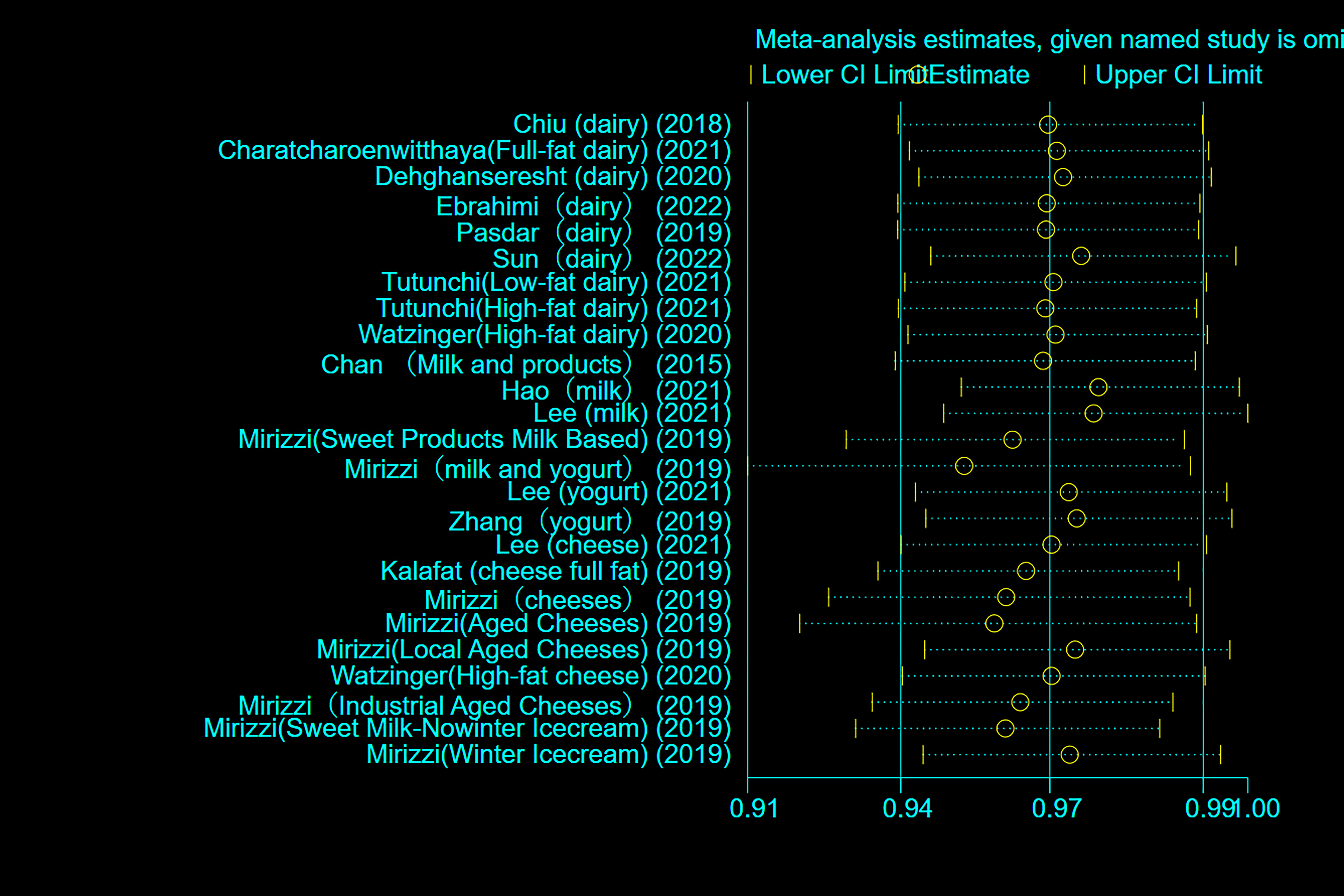

Supplement: Supplementary file 2 [file Image_1.TIFF]

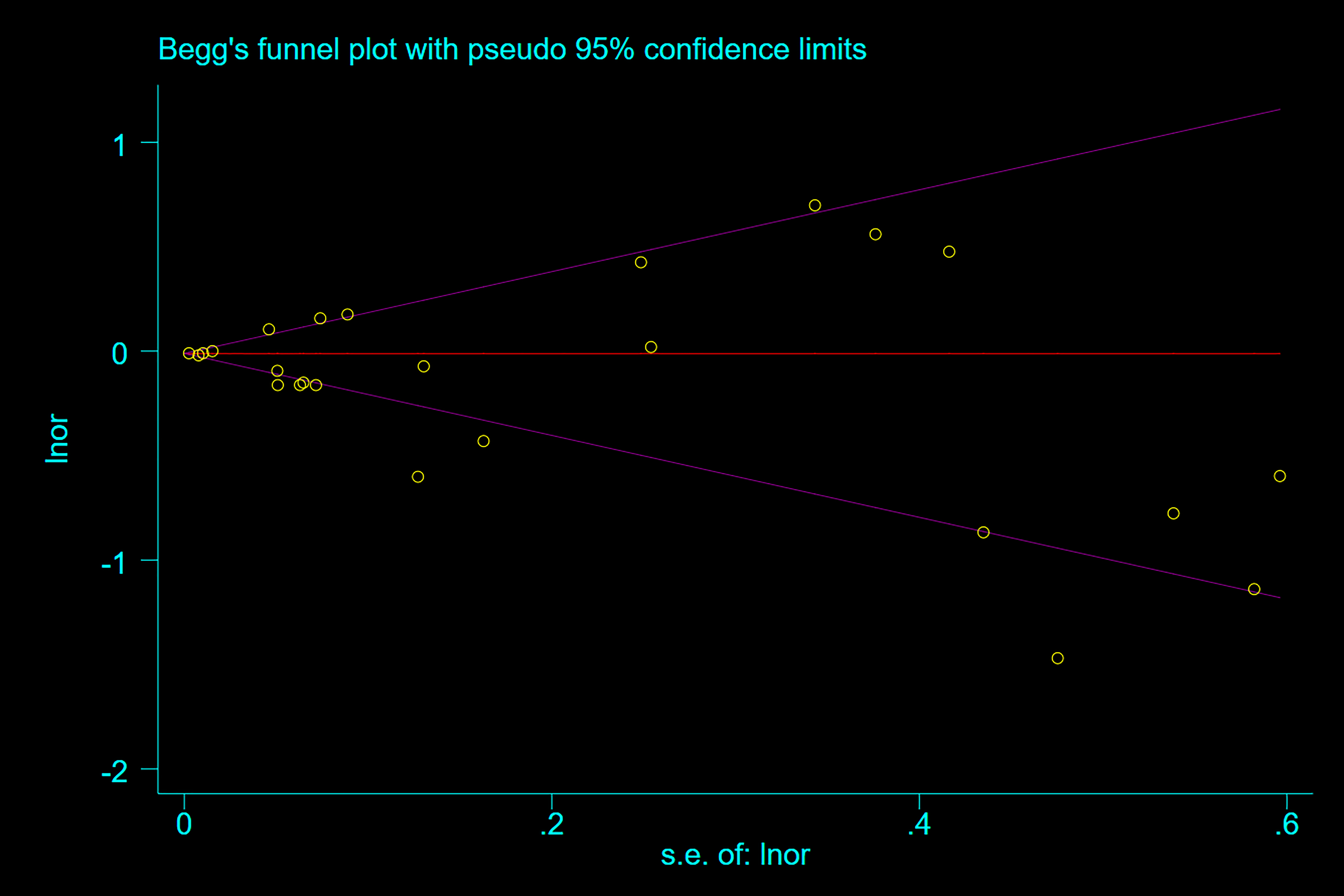

Supplement: Supplementary file 3 [file Image_2.TIFF]
