# Supplementary material for: Dairy product consumption was associated with a lower likelihood of non-alcoholic fatty liver disease: A systematic review and meta-analysis
Source: Front Nutr. 2023 Feb 22;10:1119118. doi: 10.3389/fnut.2023.1119118 (PMC9992538; doi:10.3389/fnut.2023.1119118)
Supplement: Supplementary file 4 [file Table_1.docx]

**Table S1. Meta-analysis search strategy.**

| **Database** | **Search strategy** |
| --- | --- |
| **Pubmed** | ("Dairy Products"[Mesh]) OR (Dairy Product)) OR (Product, Dairy)) OR (Products, Dairy)) OR (milk product)) OR (dairy produce)) OR (milk powder)) OR (powdered milk)) OR (dried milk)) OR (whole milk)) OR (low-fat milk)) OR (skimmed milk)) OR (at-free milk)) OR (yogurt)) OR (yoghurt)) OR (cream cake)) OR (ice cream)) OR (cheese)) OR (condensed milk)) OR (concentrated milk)) OR (milk shake)) OR (butter)) OR (milk)) OR (sherbet)) OR (cream)) OR (total dairy)) OR (frozen desserts)) OR (dairy)) OR (dairy product consumption)) OR (dairy product intake)) OR (dairy intake)) OR (fermented milk)) OR (kefir)) OR (low-fat dairy)) OR (low-fat total dairy)) OR (full-fat total dairy)) OR (full-fat milk)) AND (((("Liver Neoplasms"[Mesh]) OR (Neoplasms, Hepatic) OR (Neoplasms, Liver)) OR (Liver Neoplasm)) OR (Neoplasm, Liver)) OR (Hepatic Neoplasms)) OR (Hepatic Neoplasm)) OR (Neoplasm, Hepatic)) OR (Hepatocellular Cancer)) OR (Cancers, Hepatocellular)) OR (Hepatocellular Cancers)) OR (Hepatic Cancer)) OR (Cancer, Hepatic)) OR (Cancers, Hepatic)) OR (Hepatic Cancers)) OR (Cancer, Hepatocellular))) OR (("Non-alcoholic Fatty Liver Disease"[Mesh]) OR (Non alcoholic Fatty Liver Disease) OR (NAFLD)) OR (Nonalcoholic Fatty Liver Disease)) OR (Fatty Liver, Nonalcoholic)) OR (Fatty Livers, Nonalcoholic)) OR (Liver, Nonalcoholic Fatty)) OR (Livers, Nonalcoholic Fatty)) OR (Nonalcoholic Fatty Liver)) OR (Nonalcoholic Fatty Livers)) OR (Nonalcoholic Steatohepatitis)) OR (fatty liver) OR (Nonalcoholic Steatohepatitides)) OR (Steatohepatitides, Nonalcoholic)) OR (Steatohepatitis, Nonalcoholic) OR (Steatohepatitis, Nonalcoholic) |
| Web of Science | ＃1: TS=（Non alcoholic Fatty Liver Disease OR Nonalcoholic Fatty Liver Disease OR Fatty Liver, Nonalcoholic OR Fatty Livers, Nonalcoholic OR Liver, Nonalcoholic Fatty OR Livers, Nonalcoholic Fatty OR Nonalcoholic Fatty Liver OR Nonalcoholic Fatty Livers OR Nonalcoholic Steatohepatitis OR Nonalcoholic Steatohepatitides OR Steatohepatitides, Nonalcoholic OR Steatohepatitis, Nonalcoholic OR liver neoplasms OR Neoplasms, Hepatic OR Neoplasms, Liver OR Liver Neoplasm OR Neoplasm, Liver OR Hepatic Neoplasms OR Hepatic Neoplasm OR Neoplasm, Hepatic OR Hepatocellular Cancer OR fatty liver OR Cancers, Hepatocellular OR Hepatocellular Cancers OR Hepatic Cancer OR Cancer, Hepatic OR Cancers, Hepatic OR Hepatic Cancers）  ＃2: TS=(Dairy Products OR Dairy Product OR Product, Dairy OR Products, Dairy OR milk product OR dairy produce OR milk powder OR powdered milk OR dried milk OR whole milk OR low-fat milk OR skimmed milk OR fat-free milk OR yogurt OR yoghurt OR cream OR cream cake OR ice cream OR cheese OR condensed milk OR concentrated milk OR milk shake OR butter OR milk OR sherbet OR total dairy OR frozen desserts OR dairy OR dairy product consumption OR dairy product intake OR dairy intake OR fermented milk OR kefir OR low-fat dairy OR low-fat total dairy OR full-fat total dairy OR full-fat milk)  ＃3:＃1 and ＃2 |
| Scopus | ＃1: {dairy product} OR {milk} OR {yogurt*} OR {cream*} OR {cheese} OR {butter} OR {sherbet} OR {kefir} OR "total dairy" OR "ice cream" OR "low-fat dairy" OR "full-fat total dairy" OR "full-fat milk" OR "milk powder" OR "cream cake" OR "whole milk" OR "frozen desserts": TITLE-ABS-KEY＃2: {nafld} OR "nonalcoholic fatty liver disease" OR "nonalcoholic steatohepatitis" OR "liver neoplasms" OR "hepatic steatosis" OR {nash} OR "hepatic cancer*" OR "fatty liver" OR "liver neoplasm" OR "hepatic neoplasms" OR "hepatic cancer" OR "hepatocellular cancer": TITLE-ABS-KEY ＃3:＃1 and ＃2 |
