# Supplementary material for: Dairy product consumption was associated with a lower likelihood of non-alcoholic fatty liver disease: A systematic review and meta-analysis
Source: Front Nutr. 2023 Feb 22;10:1119118. doi: 10.3389/fnut.2023.1119118 (PMC9992538; doi:10.3389/fnut.2023.1119118)
Supplement: Supplementary file 5 [file Table_2.docx]

**Table S2. Assessment scale of cohort study or case-control study ^a^**

| Study | Selection | | | | Comparability | Outcome | | | Quality  score |
| --- | --- | --- | --- | --- | --- | --- | --- | --- | --- |
|  | Representativeness of the exposed cohort | Selection of the non-exposed cohort | Ascertainment of exposure | Demonstration that the outcome of interest was not present at start of the study | Comparability of cohorts on the basis of the design or the analysis | Ascertainment of outcome | Was follow-up long enough for outcomes to occur? | Adequacy of follow-up of cohorts |  |
| **Kalafati (2019)** | * | * | * | * | * | * | * | / | 7 |
| **Ebrahimi (2022)** | * | * | / | * | ** | * | * | / | 7 |
| **Sun (2022)** | * | * | / | * | ** | * | * | / | 7 |
| **Tutunchi (2021)** | * | * | * | * | ** | * | * | / | 8 |
| **Dehghanseresht (2020)** | * | * | * | * | ** | * | * | / | 8 |
| **Pasdar (2019)** | * | * | / | * | ** | * | * | / | 7 |
| **Lee (2021)** | * | * | * | * | ** | * | * | / | 8 |

^a^ The Newcastle–Ottawa Scale was used to assess the quality of studies
