# Supplementary material for: Dairy product consumption was associated with a lower likelihood of non-alcoholic fatty liver disease: A systematic review and meta-analysis
Source: Front Nutr. 2023 Feb 22;10:1119118. doi: 10.3389/fnut.2023.1119118 (PMC9992538; doi:10.3389/fnut.2023.1119118)
Supplement: Supplementary file 6 [file Table_3.docx]

**Table S3. Cross-sectional study quality assessment scale ^a^**

| Studies | QA tool criteria | | | | | | | | | | | Quality score |
| --- | --- | --- | --- | --- | --- | --- | --- | --- | --- | --- | --- | --- |
| Reference | 1 | 2 | 3 | 4 | 5 | 6 | 7 | 8 | 9 | 10 | 11 |  |
| **Chan (2015)** | Yes | Yes | Yes | Yes | Yes | Unclear | Unclear | Yes | Yes | Yes | No | 8 |
| **Chiu (2018)** | Yes | Yes | Yes | Yes | Yes | Unclear | Yes | Yes | Yes | Yes | Unclear | 9 |
| **Charatcharoenwitthaya (2021)** | Yes | Yes | Yes | Yes | Yes | No | Unclear | Yes | Unclear | Yes | No | 7 |
| **Hao (2021)** | Yes | Yes | Yes | Yes | Yes | Unclear | Unclear | Yes | Yes | Yes | Unclear | 8 |
| **Watzinger (2020)** | Yes | Yes | Yes | Yes | Yes | Unclear | Yes | Yes | Unclear | Yes | Unclear | 8 |
| **Mirizzi (2019)** | Yes | Yes | Yes | Yes | Yes | Unclear | Yes | Yes | Unclear | Yes | Unclear | 8 |
| **Zhang (2019)** | Yes | Yes | Yes | Yes | Yes | Unclear | Yes | Yes | Yes | Yes | Unclear | 9 |

^a^ The Agency for Healthcare Research and Quality was used to assess the quality of studies

*1. Define the source of information (survey, record review)

2. List inclusion and exclusion criteria for exposed and unexposed subjects (cases and controls) or refer to previous publications

3. Indicate time period used for identifying patients

4. Indicate whether or not subjects were consecutive if not population-based

5. Indicate if evaluators of subjective components of study were masked to other aspects of the status of the participants

6. Describe any assessments undertaken for quality assurance purposes (e.g., test/retest of primary outcome measurements)

7. Explain any patient exclusions from analysis

8. Describe how confounding was assessed and/or controlled

9. If applicable, explain how missing data were handled in the analysis

10. Summarize patient response rates and completeness of data collection

11. Clarify what follow-up, if any, was expected and the percentage of patients for which incomplete data or follow-up was obtained
